# Care of Children's Teeth

**Published:** 1878-06

**Authors:** 


### ARTICLE IV.

### *Care of Children's Teeth.*

BY DR. KLUMP.

[A Paper read before the Susquehanna Dental Association.]

There is, perhaps, nothing pertaining to dentistry that is of so much importance and yet so generally neglected as the care of children's teeth. Parents, from the want of proper knowledge regarding them, are somewhat excusable for this seeming neglect. But with the dental practitioner no such excuse should have any weight, though many might consciously, and some perhaps even willingly, acknowledge this ignorance regarding them. Parents are inclined to look upon the preservation of the temporary teeth with some misgivings, that it is to give the dentist employment at the expense of the parent for teeth which at best will be useful but a few years. Dentists, too, are very tardy in their duties in this respect for several apparent reasons. Childrens are at best restless and troublesome to operate for. We all find it much more difficult to operate for a child than for an adult. Operations for children often prove imperfect and unsatisfactory when the dentist has done his utmost to perform them carefully and well. But notwithstanding all the labor, anxiety, trouble and difficulty we have in operating for the little patients, we find that the bills are often reluctantly met, and, in some instances, considered as so much money thrown away. We see, then, that with all the trouble, vexations and difficulties these operations add but little to the exchequer of the dentist, and, perhaps, even less to his popularity. But, however, exhaustive and unsatisfactory such operations may prove, a well qualified conscientious dentist will not falter from his duty. He will not relax effort, and will not neglect a plain line of duty for something more popular or more remunerative. The deciduous teeth are subject to the same laws of decay as the permanent ones, and should, therefore, receive the same care and attention. To so modify the natural tendency to decay and insure a perfect set of teeth in all cases, is obviously impossible until our knowledge in this direction is vastly increased. We look forward, however, to greater improvements in this direction, and have great hope that the dentist of the future will be able to supply the proper nourishment for producing better teeth.

### ELEMENTS OF TEETH.

We find by chemical analysis that teeth are largely composed of lime, principally the phosphates and carbonates. It is these elements which give to them the extreme hardness found in the best teeth, while the softer and more defective teeth contain a much smaller proportion of these constituents. Many mothers suffer exceedingly from dental decay during gestation and lactation, which is doubtless caused by an insufficient supply of the phosphates and carbonates with which to keep up the repair and to build up new dental tissue. The experiments of Chossat prove that softening of the bones may be artificially produced in animals by withholding food from them containing the phosphate of lime. Common sense would suggest that if these materials are required they should be supplied, especially during the formative period in childhood. The mother should be supplied with food containing largely these ingredients from the beginning of gestation till birth, and even through the entire period of lactation. And when the child, from age or other cause, is denied the food provided by nature, we should supply food rich in these elements, and prohibit as much as possible the taste for food that contains these ingredients but sparingly. For nature always selects and appropriates from a superfluity what she needs. In the field the crop may suffer from the meagre quantities of the proper constituents needed in the soil, although the very constituents are not all exhausted—but supply these profusely and mark the almost immediate change. As the soil is improved to nourish the plants by supplyingt the necessary constituents, so it seems to us the blood may be improved so as to properly nourish every part of the hu man economy. It seems obvious that we should supply through the food these elements, rather than by medicinal means, on account of the greater probability of assimilation and appropriation.

### THE SECOND SET OF TEETH.

The regularity and evenness of the second set is also dependent upon the preservation and retention of the deciduous teeth. If caries is not prevented or arrested it will not only destroy the temporary teeth, but the permanent ones will also suffer from them, especially those in close proximity. It is also important to retain the diciduous teeth in good condition till they are replaced by the second set, both for the comfort and health of the child. When they are defective there is generally more or less pain produced in mastication, and then the child, from its desire to avoid pain, uses them less and less, as they grow worse, till the food is finally swallowed nearly, if not entirely, in the same condition that it is taken into the mouth. Such a habit once fully contracted is scarcely ever entirely overcome. The food then being only partially masticated, digestion is impaired, and the duties of the stomach are thus augmented; dyspepsia with all its concomitants ensues, and drags the patient through a pitiable life down to an early grave. The design of nature is to preserve the crowns of the temporary tooth till its roots are absorbed and it becomes loose and is removed to make room for its permanent successor. Could the temporary teeth always be retained in a perfect condition, it would save much of the defectiveness and irregularity of the succeeding set. Then, again, the premature extracton of the deciduous teeth frequently leaves such very terrible impressions upon the mind of the child, that in after years the permanent set are neglected and lost in consequence of the frightful associations connected with their early experience in dentistry. By carefully preserving the child's teeth we spare it much pain and suffering during mastication. We prevent the tendency of bolting the food. We prevent much of the irregularity and unevenness in the succeeding set. We prevent injury to the general health, as the immediate result of imperfect mastication. But, notwithstanding, the great necessity of preserving the temporary teeth, it is obviously more important to preserve the permanent ones. How much an even, regular set of teeth add to the comeliness of the features and the beauty of the expression? And how small any alteration in the teeth may distort the features and cause a disagreeable ugliness. The extraction of a single tooth, either for youth or adult, will produce a change in the remaining teeth. This may be so slight as to be noticeable only upon close examination, or it may be so marked as to cause a hideous deformity. It is surprising to observe how slight a change in the features of one comely and beautiful may cause a disagreeable, ugly expression. A great many of the most disagreeable deformities are caused by injudicious extraction, and often, too, by men who are otherwise worthy practitioners.

#### OTHER CAUSES.

And then there are other cases, although not actually caused by the operator, which might have been prevented or easily corrected, without any of the ordinary regulating appliances by judicious and timely extraction. It does seem as if this subject was pressing itself upon the notice of the profession with such alarming frequency as to demand our most thoughtful attention. Should there be any doubt in regard to its seriousness and importance, it is only necessary to examine carefully every case of irregularity with a view of ascertaining the cause, to convince the most indifferent or skeptical. Upon examination you will observe where the upper front teeth strike upon or inside of the lower teeth, the irregularity is often caused by the injudicious extraction of the superior first molars; or you may have an apparent protrusion of the upper teeth, caused by the extraction of the inferior first molars. The front teeth may lean to one side, either or both jaws be contracted. Bicuspids and molars leaning backward or forward, either or both, and other equally prominent malformations may all be traced to this evil. But we sincerely protest against the indiscriminate extraction of any tooth, however offensive or troublesome. Every case should first be carefully examined and the effect upon the remaining teeth duly considered. We should learn to understand the laws of nature in the structure and development of the teeth, and use them to assist us in producing the symmetry and beauty of the features, and in preventing and correcting irregularities in their incipiency.

